# The Yellow Flag: Quarantine and the British Mediterranean World, 1780–1860

**DOI:** 10.3201/eid2708.210997

**Published:** 2021-08

**Authors:** Rebecca Wurtz

**Affiliations:** University of Minnesota School of Public Health, Minneapolis, Minnesota, USA

**Keywords:** quarantine, Britain, shipping, travel

In reading the Centers for Disease Control and Prevention’s February 2021 order (*1*) requiring passengers to wear face masks while on conveyances, I learned a new concept: “free pratique,” the permission granted by a government to an international vessel (ship, plane, or other) to disembark its passengers once it has been deemed clear of contagion. That same day, opening *The Yellow Flag: Quarantine and the British Mediterranean World, 1780–1860*, by Alex Chase-Levenson (Figure), I encountered the concept again, in the same context: 18th-century travelers freed from quarantine’s constraints were considered to be in “free pratique” (p. 15). History may not repeat itself, but it does rhyme.

**Figure:**
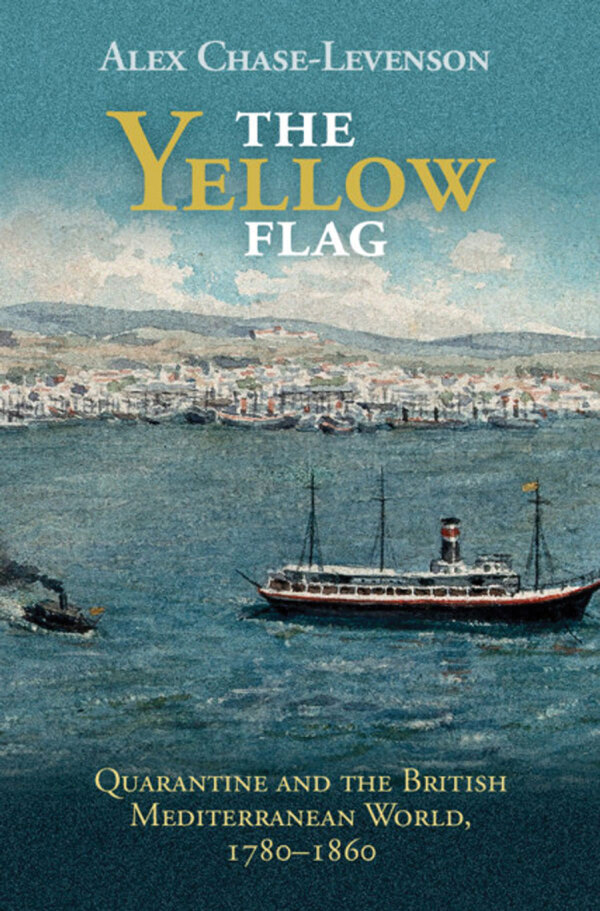
The Yellow Flag: Quarantine and the British Mediterranean World, 1780–1860

When we think of the history of how one nation defines itself in relation to others (e.g., borders, trade, diplomacy), we do not usually include communicable disease control in the list. *The Yellow Flag* describes the administrative practices requiring quarantine of travelers and goods arriving in ports by ship (and, in some cases, at land crossings) from places considered “foul.” Ships flying the yellow flag were deemed “plague smitten” and their passengers and cargo were subject to a range of complex, changeable rules.

Chase-Levenson focuses on Britain, a country perceived in the early 1800s as still at the geographic, political, and economic edge of Mediterranean power and politics. The edge case is illuminating. The anxieties accompanying increasing “globalism” in the 18th century (even if “global,” for the purposes of *The Yellow Flag*’s era, meant the Mediterranean basin) so perfectly rhyme with those of the 21st: efforts to exclude or expedite the passage of persons and goods, but also collaboration among health and medical experts across many countries and jurisdictions to form coherent and defensible policy. Scientists, bureaucrats, and citizens stumbling in understanding cause and effect, prevention and efficacy. The fervor with which anticontagionists (persons who denied that the diseases of concern were spread from person to person) defended their position, including an account of an anticontagionist who injected himself with blood from a plague victim to disprove the theory of contagion, with tragically predictable results. Social media, in the form of mass-produced pamphlets, warning of secret plans, conspiracy, and perfidy. The performative aspects of infection control, such as fumigating with a few pinches of nitre on a tray of charcoal.

Chase-Levenson lays out these stories clearly and systematically. The narratives of individual travelers, written while in quarantine, were particularly entertaining, although the depth of detail may engage the historian more than the epidemiologist. I have but one criticism: the index seems incomplete. For example, the term “pratique” is not included. That is not the author’s fault, but the publisher’s.

The purpose of reading history has, to me, always been to both bring our ancestors closer—they were truly just like us in their triumphs and tragedies—and to learn what we can from their experiences to illuminate our present day. When future historians examine the COVID-19 pandemic, what will they marvel at? And shake their heads at? I have thought about that again and again during the past year and a half. Let us hope the historians are as insightful, and the results as readable, as Chase-Levenson’s inquiry.

## References

[1] Requirement for persons to wear masks while on conveyances and at transportation hubs. 86 Fed. Reg. 8025, February 3, 2021 [cited 2021 May 11]. https://www.federalregister.gov/documents/2021/02/03/2021-02340/requirement-for-persons-to-wear-masks-while-on-conveyances-and-at-transportation-hubs

